# “F*ck It! Let’s Get to Drinking—Poison our Livers!”: a Thematic Analysis of Alcohol Content in Contemporary YouTube MusicVideos

**DOI:** 10.1007/s12529-016-9578-3

**Published:** 2016-09-06

**Authors:** Jo Cranwell, John Britton, Manpreet Bains

**Affiliations:** Division of Epidemiology and Public Health, Clinical Sciences Building, Nottingham City Hospital, UK Centre for Tobacco and Alcohol Studies, University of Nottingham, Nottingham, NG5 1PB UK

**Keywords:** Alcohol, YouTube music videos, Thematic analysis, Alcohol policy, Adolescent role modelling

## Abstract

**Purpose:**

The purpose of the present study is to describe the portrayal of alcohol content in popular *YouTube* music videos.

**Method:**

We used inductive thematic analysis to explore the lyrics and visual imagery in 49 UK Top 40 songs and music videos previously found to contain alcohol content and watched by many British adolescents aged between 11 and 18 years and to examine if branded content contravened alcohol industry advertising codes of practice.

**Results:**

The analysis generated three themes. First, alcohol content was associated with sexualised imagery or lyrics and the objectification of women. Second, alcohol was associated with image, lifestyle and sociability. Finally, some videos showed alcohol overtly encouraging excessive drinking and drunkenness, including those containing branding, with no negative consequences to the drinker.

**Conclusion:**

Our results suggest that YouTube music videos promote positive associations with alcohol use. Further, several alcohol companies adopt marketing strategies in the video medium that are entirely inconsistent with their own or others agreed advertising codes of practice. We conclude that, as a harm reduction measure, policies should change to prevent adolescent exposure to the positive promotion of alcohol and alcohol branding in music videos.

## Introduction

Adolescent alcohol consumption, including binge drinking, is a significant health problem in the UK [1–3]. Alcohol consumption is related to the risks of cancer, cardiovascular and liver diseases, and binge drinking worsens all of these risks [4]. High alcohol consumption among young people represents a significant public health problem because it is associated with deleterious effects such as criminal behaviour [5, 6], unprotected sexual intercourse [7], is a risk factor for dependence in later life [8, 9] and progression to illicit drug use [10, 11]. In the UK, 11 % of 15–16 year olds out of a sample of 2000 had had sex under the influence of alcohol and regretted it and almost 10 % of boys and around 12 % of girls reported having unsafe sex after drinking alcohol [12].

Risk factors for use in adolescence not only include a range of family and personality characteristics [13] but also include exposure to alcohol content in advertising, films, television and music videos [14–25]. The effects of media exposure are consistent with theoretical perspectives from social learning theory, which suggests that behaviour is learned through the observation of role models, especially ones that have valued personal attributes (e.g. celebrities) and respondent conditioning theory (RCT) [26, 27]. RCT in alcohol advertising might, for example, comprise presenting a brand (the conditioned stimulus, originally neutral) a number of times with images of attractiveness (the unconditioned response), to form a positive reaction or feeling that is then also generated by exposure to the brand (the conditioned response). However, these types of “hyperdermic” models of influence have been criticised by Gill [28] as naive, reductionist and a simplistic understanding of media influence [28]. Gill also argues that young “media-savvy consumers” in a postfeminist and neoliberalist era are perhaps too sophisticated to be “got at” by adverts that explicitly appear to be selling directly to them [28]. Similarly, D’Orio (1999) suggests that modern youth may not be responsive to traditional forms of marketing or promotion and that novel methods may be more effective [29]. Ruddock (2012) argues that alcohol advertising strategies, especially using social media, that use social communication as a form of advertising as opposed to official advertisements are a move beyond traditional advertising strategies that Gill alludes to [30]. This below-the-line alcohol promotion that exploits existing cultural practices in social media suggests that advertising messages might be harder to reject by the consumer. For example, the Facebook pages of Smirnoff and Victoria Bitter have been found to promote excessive alcohol consumption by asking people to post comments about their drinking behaviour [31] and researchers suggest that by sharing these types of messages, posters on social media sites such as Bebo, MySpace and Facebook are building “intoxigenic social identities” that function to normalise drinking alcohol [31–34]. Further, Smirnoff has been found to credit alcohol consumption with social and sexual prowess with photos of young male fans in bars and clubs surrounded by attractive young women in close-fitting Smirnoff branded dresses being posted on their Australian Facebook page [31].

In music videos and song lyrics, alcohol has been associated with sexual activity [35, 36], partying, vehicles, other drugs, wealth/luxury (lyrics) [37], drunkenness, crime and violence (lyrics) [36] and humour (music videos) [38]. Featured alcohol brands tended to be expensive cognacs such as Remy Martin™ or Hennessy™ or other spirits [36, 39]. Advertising and promotion of alcohol in the UK are not only largely self-regulated by the drink industry but are also covered by Advertising Standards Authority (ASA) and the Portman Group (PG) codes of conduct [40, 41]. These codes are in place as a reference framework for the alcohol industry to check that their alcohol brands are not marketed to those under the age of 18 years and that they are promoted in a socially responsible manner. Alcohol promotion in new media is subject to these codes that aim to avoid the promotion of drinking to a youth audience; however, social media websites have been identified as key alcohol marketing channels where alcohol brands interact with young members in novel and strategic ways. For example, Brodmerkel and Carah ( 2013) posit that alcohol brands use Facebook to “manage the mediation of drinking culture in a way that challenges existing regulatory codes by prompting consumers to say things that brands are prohibited from saying” (p. 274) [31].

The development of social media has made music more accessible to young people than ever before. In April 2011, music videos accounted for 30 % of the top 10 most viewed channels on the social networking site *YouTube* [42]. Further, YouTube, which dominates the music video-sharing market in the UK, is particularly popular among 12–17 year olds [42] and is the most popular medium for accessing music by American youth [43], rendering access via traditional media such as MTV and CDs outdated. Music is ubiquitous in adolescent life, and research suggests that young people do watch music videos because they represent aspects of lifestyles that can be incorporated into their own lives (28). Research also suggests that music behaves as an adolescent identity symbol and secures youth subcultures [44]. For example, Clay [45] found that young girls emulate women in hip-hop videos in order to authenticate their own Black identity [45]. This suggests that lyrics and imagery allow audiences to associate brands with particular kinds of lifestyles, artists (e.g. musicians) and culture (e.g. hip-hop). Even brief exposure to music videos could influence behaviour [46]. Brands are also a key feature of a hip-hop music artist’s identity, and they will often feature them in their music often offering marketers brand placement in their music as “bait” for lucrative deals [47].

However, since little is known about the alcohol content in music videos on social media such as YouTube, we have recently quantified alcohol content in a selection of the videos of popular UK songs where alcohol content occurred in 45 % (49) of videos in our sample [48] and provided evidence that, per capita, British adolescents receive approximately four times more alcohol and tobacco-related messages from these videos than British adults [49]. In order to bring the research up to date, we now present an inductive thematic analysis of the content that we found, both lyrical and visual, which is missing in the existing literature. The research questions guiding this study are as follows:

1. How is alcohol portrayed in music videos?
2. Are UK alcohol industry advertising codes of practice being violated?

## Method

We searched all songs listed in the Official Singles Chart UK Top 40 [50] and the Vodafone Big Top 40 music chart [51], published on the 12 Sundays (Sundays being the day on which changes in the music charts are reported) from 3 November 2013 to 19 January 2014 [48]. Of the 130 songs identified from these two chart lists, we then identified companion YouTube music videos for 110. All videos were coded for the presence or absence of alcohol in the following categories:

- *Alcohol use*: actual consumption of an alcoholic drink by any character.
- *Implied alcohol use*: open bottles of or glasses appearing to hold alcoholic drinks, drunken behaviour or other appearance implying alcohol consumption but without actual use.
- *Alcohol paraphernalia*: bottles, glasses or other materials associated with alcohol (for example, a shot of a bar containing alcohol bottles and glasses) without actual or implied use.
- *Alcohol brand appearance*: clear and unambiguous alcohol branding on a product consumed or otherwise visible in the scene, or in advertisements, logos or other recognisable branded materials.

The present analysis reports the findings of the qualitative arm of our quantitative content analysis and was carried out on the 49 videos which contained alcohol content. A detailed account of the original quantitative coding procedure has been published elsewhere [48]. All song lyrics were obtained from two online websites: www.azlyrics.com and www.songlyrics.com.

## Procedure and Data Analysis

We conducted an inductive thematic analysis as described by Braun and Clarke [52] and followed established steps commonly used to analyse qualitative data [53]. First, in order to prevent possible researcher bias and ensure analytic rigour, the music videos were subjected to multiple viewings independently by two experienced qualitative researchers (raters: authors JC and MB). This intense immersion process aided familiarisation with the content and identification of patterns within the data. Second, scenes containing salient alcohol-related content (visual or lyrical), and descriptions of their context, were systematically coded independently by both raters. Similar to previous research using multilevel data in video games, the lyrics were linked to the visual data files rather than being conceptually separated [54]. For example, during each 10-s interval where alcohol visually occurred, we wrote down a detailed summary of what was occurring and then analysed the data also in relation to lyrical content (vice versa for the verbal data). This process was tedious and time consuming, but we agree with others that it was a crucial step that made the data accessible for both researchers for analysis and it also helped in contextualising the data [54]. Third, broad codes were attached to the content of ten videos and grouped into potentially relevant themes with corresponding sub-themes by each rater. The lists of themes were then jointly reviewed to check for similarities between each rater list and if the codes represented them appropriately. This allowed discussion, clarification and refinement of the specific nature of the themes. Data within each subcategory were re-examined, and in the event of any apparent contradiction to any of the themes, the indexing process was re-evaluated by both raters to either include or exclude the contradiction and the theme was redefined as appropriate. This process was repeated on a second set of ten videos and again on any remaining videos until all was coded. Finally, the themes were presented to the school’s research group (approximately 20 researchers) to check that they were coherent and that content represented them appropriately. Minor revisions were made to the theme labels, where appropriate.

## Results

Thirty-seven out of 49 of the music videos were included in the final themes. Twelve videos were not included in the final themes as the data did not adequately represent them or they included quick appearances of paraphernalia that were difficult to contextualise (e.g. a bottle of wine visible in the background on a kitchen worktop). Three core themes were generated from the inductive analysis, associated with alcohol use: *sexualised imagery or lyrics and the objectification of women*, *alcohol and image*, *lifestyle and sociability* and *drinking to excess*. Table 1 provides details of the videos included and examples of content, including branding.

**Table 1:**
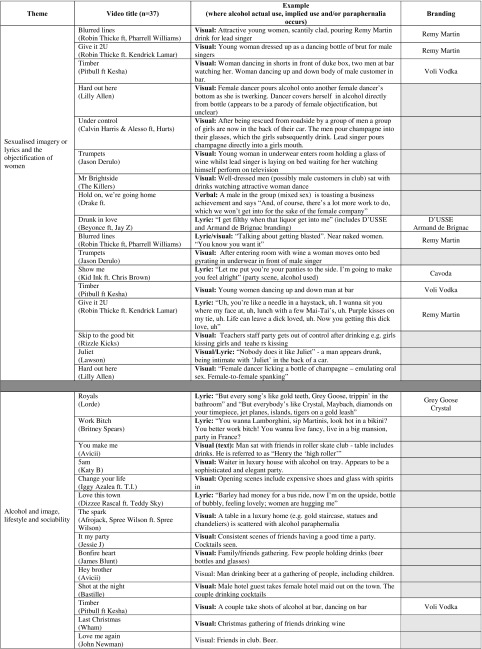

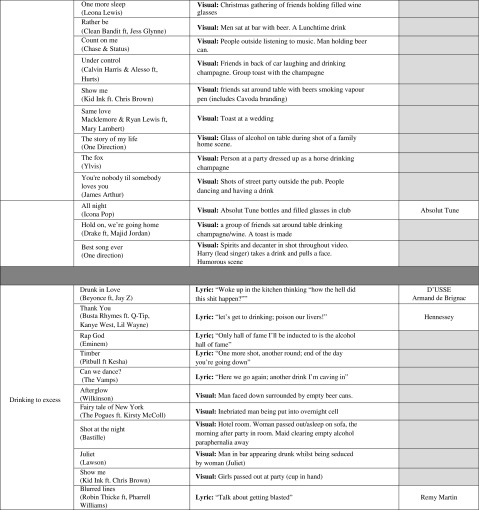
Themes including video title and branding content

Greyed-out boxes denote no branding present in music video

### Sexualised Imagery or Lyrics and the Objectification of Women

This theme highlights that some music videos portrayed women in highly sexualised ways with a focus on women and women’s body parts and that alcohol was used as a prop or key element in these portrayals. The video “Blurred lines” by Robin Thicke includes women in underwear dancing around the male singers, all of who are fully clothed. At one point, the lead singer pours and drinks branded Remy Martin™ cognac, while the lyrics repeat the phrase: “You know you want it”, the implication of which is evident in a subsequent lyric: “I’ll give you something big enough to tear your ass in two”. In another video “Give it 2U”, by the same artist, which again includes Remy Martin™ branding, a young woman emulates pouring alcohol from a giant cut-out shaped like a champagne bottle into his mouth, while other women dance around him, one wearing a thong, followed by his lyric: “I want to give it to you tonight” and “Uh, you’re like a needle in a haystack, uh. I wanna sit you where my face at, Uh. Lunch, with a few Mai-Tai’s, uh. Purple kisses on my tie, uh. Life can leave a dick loved, uh. Now you gettin’ this dick, love, Uh”. In “Timber” by Pitbull, one scene depicts a young woman dancing up and down the body of a man who is a customer in the bar; another depicts a man with a beer in his hand, watching women dance for him. The scenes are interspersed by both visual and lyrical advertising of the Voli™ vodka brand. The video “Show me” by Kid Ink and Chris Brown, which contained Cavoda™ vodka branding, included images of young women grinding against men and girls kissing each other next to another woman who had passed out at a party. In “Skip to the good bit” by Rizzle Kicks, a teacher’s staff party becomes out of control with the portrayal of sexually suggestive scenes such as girls licking girls and teachers kissing (however, most content appeared to portray compulsive heterosexuality).

Interestingly, some of these alcohol-related behaviours represent a mixture of both traditional and non-traditional roles of masculinity and femininity. Alcohol is linked strongly with expressing masculinity [55], and the examples that we highlight appear to suggest that it was used to conceptualise male power and was associated with the explicit objectification of women. In some videos, women were portrayed as designed to entertain the male onlooker who is drinking and which also attributes alcohol to male sexual prowess or success. Railton and Watson (2011) conclude that there is a unity across versions of masculinity presented in music videos, and this unity represents global masculine values [56]. Whilst the traditional roles (e.g. drinking is a male behaviour and female drinking is masculine or deviant) are consistent with previous research on alcohol drinking behaviours in magazines and music videos [56, 57], we also found evidence of alcohol use that challenged traditional notions of femininity.

Traditional femininity expectations generally call for lower alcohol consumption than men and do not link it to drinking, and excessive use of alcohol by women is viewed as masculine or deviant [58–62]. However, the scenes in, for example, “Show me” by Kid Inc (featuring Chris Brown), which suggests excessive alcohol use by a female, suggest that these expectations are being challenged through music videos or that they reflect changing female culture or a “new femininity” in relation to drinking. Recent research suggests that young women are “breaking traditional codes of femininity” by becoming frequently intoxicated and associate drinking with pleasure and fun, which has consequences for risky behaviours whilst drunk [61]. Arguably, images of women drinking to get drunk in music videos may therefore serve to normalise binge drinking in young women.

Notions of female passivity and objectification are banished in the video “Drunk in love” by Beyoncé (featuring Jay-Z). This video includes brand references to Armand de Brignac™ champagne and D’USSE™ cognac in association with Beyoncé’s sexual activity and getting out of control after drinking. For example: “I’ve been drinking, I’ve been drinking; I get filthy when that liquor get into me”. Gill [28] refers to this “feisty”, “sassy” and “sexually agentic” female type as “the midriff”—a female who, rather than being the object of the male gaze, is the active subject, one which is powerful and confident [28]. Further, Beyonce’s video links getting drunk with “getting filthy,” reinforcing the notion that women being sexually active (or assertive) are “dirty” and seemingly not adhering to traditional femininity standards. The message appears to be that transgressing from these standards is possible for women through the excessive consumption of alcohol. The extent to which these portrayals might influence youth drinking remains unclear. However, research on alcohol in television commercials does suggest that adolescents exposed to role characterisations of media drinkers, who identify with the drinker roles and value their traits, may be more likely to imitate their behaviour [63]. Therefore, during sexual maturation, adolescent exposure to celebrities condoning the use of alcohol combined with sexual activity may pose an additional risk for adolescent health and for identity development in both boys and girls.

The PG alcohol code states that alcohol should not have “any association with sexual activity or sexual success” [64], and section 18.5 of the ASA codes of practice states “Marketing communications must neither link alcohol with seduction, sexual activity or sexual success nor imply that alcohol can enhance attractiveness” [40], yet we found links between alcohol and attractiveness and/or sex in the videos containing brands such as Timber (Voli™), Show me (Cavoda™), Blurred lines and Give it 2U (Remy Martin™) and, particularly, in Drunk in love, which features the D’USSE™ brand owned by Bacardi USA, Inc [65]. This indicates that alcohol companies do want young people to have positive first impressions of their alcohol brands even before they start to drink.

### Alcohol and Image, Lifestyle and Sociability

First, this theme shows that alcohol was portrayed as a marker of success and wealth. Amongst other brands, the lyrics in “Royals” by Lorde that include branded Grey Goose™ vodka and Crystal™ champagne portray brands as being things some people appear to be preoccupied with: “But every song’s like gold teeth, Grey Goose, trippin’ in the bathroom. …. But everybody’s like Cristal, Maybach, diamonds on your timepiece, jet planes, islands, tigers on a gold leash…”. Examples of associations between alcohol and a seemingly ostentatious lifestyle include “Work bitch” by Britney Spears: “You wanna Lamborghini, sip martinis, look hot in a bikini? You better work bitch! You wanna live fancy, live in a big mansion, party in France?” Other associations with alcohol content included an upmarket club, high-end alcoholic beverages, large luxurious houses and imagery containing the word “luxury”. Pernod Ricard’s brand “Absolut Tune”™ [66, 67] was also heavily promoted alongside images of attractiveness, fashion and glamour in the video “All night” by Icona Pop. Pernod Ricard UK is also a code signatory of the PG alcohol codes.

Second, positive representations of alcohol in association with socialising, dancing, partying and relaxing with friends were identified. However, one video (“Of the night” by Bastille) contained a deficit reason for drinking [63], whereby alcohol was loosely associated with murder, suicide and crime. Another negative reference was also found in “Free” by Rudimental featuring Emilie Sandé: “I drink a little more than recommended, “cause this ain’t exactly what my heart expected”. However, other potentially negative consequences of alcohol use were negated by being portrayed in a light-hearted and ambivalent manner. Scenes of people relaxing around a table with food and friends or at outdoor gatherings with friends, family or neighbours portrayed alcohol consumption as innocuous, normal and enjoyable in “One more sleep” by Leona Lewis and “You’re nobody ‘til somebody loves you” by James Arthur. Alcohol was used to mark an occasion; for example, popping a champagne cork at a wedding (“Same love” by Macklemore), “raising a glass of wine for the last time” (“I see fire” by Ed Sheeran) and celebrating a successful business achievement were observed (e.g. “Cheers! and Salut!” in “Hold on we’re going home” by Drake).

Importantly, lifestyle or image-oriented alcohol promotion is particularly appealing to adolescents [68, 69] and creates more favourable attitudes towards specific alcohol brands in comparison to product or informational advertising [70]. Bonnie and O’Connell suggest that these types of lifestyle marketing practices have the potential to embed alcohol brands into the lifestyles of consumers and that a set of values is also being marketed [71]. Thus, the alcohol brand becomes strongly associated with these values by creating an experience that consumers directly identify with the product. Further, brand marketing using lifestyle themes has the potential to “create” culture, one of which the consumer wants to join and which the alcohol brand is the price of admission [71, 72]. Likewise, we argue that even generic depictions of drinking that do not overtly contain sales messages, but which symbolise success, may too work in a similar way. Further, exposure to positive depictions of alcohol use with the absence of negative consequences may increase the likelihood of experimentation with alcohol [26] and even the seemingly innocuous examples of drinking in social gatherings may “condition” implicit favourable presumptions about drinking that influence behavioural responses [63].

### Drinking to Excess

Whilst some videos presented seemingly innocuous drinking in social gatherings, others included substantial use of behavioural and verbal messages that encourage drinking to excess or drunkenness, again without acknowledging any negative consequences. Examples include lyrics such as “One more shot, another round, end of the day you’re going down” (Timber by Pitbull), the implication of which is “drinking until you drop”. Eminem raps “Only Hall of Fame I’ll be inducted to is the Alcohol Hall of Fame” in the song “Rap God”, which suggests that excessive drinking is a “badge of honour”. The song Drunk in love by Beyoncé had multiple references to overconsumption throughout. Moreover, alcohol was portrayed as a key factor facilitating changes in normal behaviour, such as a loss of inhibitions: “Here we go again, another drink I’m caving in, and stupid words keep falling from my mouth” (“Can we dance?” by The Vamps).

Whilst it is likely that some alcohol brand imagery is included in music videos without any involvement of or promotion by the manufacturer, our findings demonstrate that the alcohol industry is not abiding by PG and ASA industry codes. For example, the marketing code of practice of the drink company Diageo, which distributes the cognac brand Hennessy™, states “We will not associate our brands with the attainment of or ‘rites of passage’ to adulthood” [73], yet, Hennessy™ branding features in the video “Thank you” alongside the lyric “Please don’t throw up, hold your liquor; grow up!”. Diageo also state “We will not depict people drinking heavily or rapidly, or in a state of intoxication, nor imply that such behaviour is attractive or appropriate” [74]. However, the Hennessy™ brand occurs alongside lyrics clearly advocating drinking to excess: “Let’s get to drinking—poison our livers” and “Drink gallons of litres” in the same song which promotes pro-drinking attitudes. The drink company Remy Cointreau is a code signatory to the PG code of practice yet appears to breach the code which states that alcohol promotion should not “encourage illegal, irresponsible or immoderate consumption, such as drink-driving, binge-drinking or drunkenness” [75]. Drinking as a manly behaviour has also been observed in beer commercials suggesting that “real men drink” [76]. Further, Remy Cointreau’s brand Remy Martin™ [77] is featured alongside lyrics such as “talk about getting blasted” in Blurred lines.

## Conclusion

This study analysed both the visual and lyrical elements of the music and has focussed on YouTube, which is a global mass communication channel. Our findings therefore extend current research by looking at video content that has massive global reach to a youth audience. Our study was limited to the analysis of top 40 records over one 12-week period, so our findings may not represent content in videos from less popular records or at other times. This inductive analysis of alcohol portrayal in the content of YouTube music videos generated three prominent themes associated with both generic and branded alcohol: sexualised imagery or lyrics and the objectification of women, positive image or lifestyle-oriented attributes such as wealth and luxury and sociability, and drinking to excess. Our findings are consistent with previous studies that found that alcohol in both music videos and lyrics is associated with sexual activity [35, 36], wealth/luxury (lyrics) [37] and drunkenness (lyrics) [36], suggesting that they are broadly representative.

## Breaches of Advertising Codes of Practice and Music Artists as Brand Ambassadors or Co-Owners

Targeting a youth market is a key strategy for the drink industry, and new media are being adopted increasingly as a channel to communicate new drink brands to a youth market [78]. Specifically, alcohol placement in music videos is argued to be a deliberate industry marketing strategy [44]. We have previously reported that estimated exposure to music video content among British adolescents is proportionately high compared to British adults [49]. Music videos are thus a potentially highly effective means of advertising brands and consumption behaviours to young people. From both a policy perspective, the role of the alcohol industry in allowing its brands to be used, if not actually paying to do so, also needs to be considered. The overt use of celebrity endorsement or brand ambassadors of alcohol products is also a matter for concern and one, which again appears to contravene voluntary codes of practice. The music artists involved in this direct promotion in our video sample include Robin Thicke, who is described as a “brand ambassador” for Remy Martin™ [79]; Jay Z, who is a brand ambassador for D’USSE™ [80]; and Icona Pop who is the “brand face” of Absolut Tune™ [67]. Music artists are frequently used as brand ambassadors; Busta Rhymes increased the sales of Courvoisier cognac through his song “Pass the Courvoisier” [81], Jay-Z is associated with Ace of Spades cognac, and rapper P Diddy refers to himself as “Ciroc Obama” (vodka) [82]. Pitbull is described as a “shareholder” in the Voli™ vodka drink company [83], and American rapper Nicki Minaj co-owns Myx Infusions, which features in the music videos “Anaconda” and “High School”, and rapper Ludacris co-owns Conjure Congacs [82]. Vernallis (2004) argues that music videos represent the desire of the artists to forge a “brand” and therefore cannot be equated to advertising. Some alcohol content is likely to arise independently from any industry-driven marketing or advertising; however, because some of the music artists featured in our sample are brand ambassadors for the alcohol industry or own their own brands, we argue that, by masquerading as something other than advertising, this medium has even greater potential to persuade and influence.

## Recommendations

Unlike television and film, music videos are not classified according to age suitability and the video makers are not required to provide viewer advice on content relating to addictive substances such as alcohol. Because our findings that YouTube music videos include alcohol content that is associated with enhancing sexual attractiveness and luxury lifestyles, promoting excessive use and generally normalising drinking suggest that this policy should change. Alternatively, the music industry should implement standards to reduce the impact of both generic and branded alcohol content by working with music artists and alcohol companies particularly to reduce brand advertisement, both lyrical and visual, and content that condones drinking to excess. Certainly, our findings suggest that several alcohol companies adopt marketing strategies in the video medium that contravene their own advertising codes of practice, suggesting that more rigorous and enforceable codes are required. Moreover, the current PG and ASA codes are failing to police how third parties use and portray alcohol brands in content that is not developed, sponsored or distributed by the alcohol companies themselves. The UK Department of Health’s Public Health Responsibility Deal Alcohol Network [84] is thus evidently failing in this respect. Finally, we recommend that adolescents are educated in media literacy in order to reduce impact of the video content and, thus, the potential for modelling behaviours that could be detrimental to their health.
